# Recovering From Stevens-Johnson Syndrome and Toxic Epidermal Necrolysis

**DOI:** 10.1001/jamadermatol.2025.4345

**Published:** 2025-11-12

**Authors:** Michelle D. Martin-Pozo, Elizabeth A. Williams, Kemberlee R. Bonnet, Benjamin H. Kaffenberger, David G. Schlundt, Elizabeth J. Phillips

**Affiliations:** 1 Center for Drug Safety and Immunology, Vanderbilt University Medical Center, Nashville, Tennessee; 2 Department of Psychology, Vanderbilt University, Nashville, Tennessee; 3 Department of Dermatology, The Ohio State University Wexner Medical Center, Columbus; 4 Institute for Immunology and Infectious Diseases, Murdoch University, Perth, Australia

## Abstract

**Importance:**

Stevens-Johnson syndrome and toxic epidermal necrolysis (SJS/TEN) survivors experience substantial long-term sequelae. Research on physical symptoms experienced during acute hospitalization is well documented, but limited studies have been completed on the long-term biopsychosocial effects of SJS/TEN, particularly from the patient’s perspective.

**Objective:**

To increase the understanding of the long-term complications of SJS/TEN.

**Design, Setting, and Participants:**

This qualitative investigation was completed from within a community-based study, the SJS Survivors Study, using a semistructured, in-depth interview guide to query participants about their SJS/TEN experience postdischarge from the hospital. Interviews took place by phone from July 2021 through August 2023. This study included adults who experienced SJS/TEN within the United States.

**Main Outcome and Measures:**

A biopsychosocial theory-based framework and hierarchical coding system were utilized to understand the long-term life impacts of survivors of SJS/TEN.

**Results:**

The 29 participants, aged 26 to 76 years, were 66% female and 69% White and had experienced SJS/TEN from a wide range of drugs. Patients experienced support while in the hospital, but once discharged, felt isolated and without support to understand the potential sustained impacts of SJS/TEN in their lives and the lives of their family members. Patients experienced ongoing biological symptoms, such as skin issues, debilitating visual impairment, blindness, and lack of functional autonomy. Psychological impacts included symptoms of anxiety, obsessive thinking, flashbacks, and depression. Socially, some survivors expressed a sense of abandonment and described negative impacts on their careers. Survivors also expressed frustration and isolation with having to navigate posthospital care alone. There was a lack of preemptive discharge education and SJS/TEN-specific planning. Lack of physician knowledge about SJS/TEN was particularly noted and survivors turned to the internet for guidance instead of receiving direction from their physicians. Medical distrust among survivors was frequently noted.

**Conclusions and Relevance:**

The findings highlight the need for postdischarge care coordination among patients and their primary physicians, including mental health support. This care coordination should be arranged prior to discharge to ensure the availability of adequate support and optimal health outcomes. It is essential that clinicians and researchers prioritize the understanding of long-term sequelae of SJS/TEN and improve current discharge education and protocols for patients and their families.

## Key Points

**Question:**

What are the long-term mental and physical health effects of Stevens-Johnson syndrome and toxic epidermal necrolysis (SJS/TEN)?

**Findings:**

In this qualitative study, survivors of SJS/TEN faced a myriad of biological, psychological, and social impacts associated with SJS/TEN. Survivors expressed medical distrust, particularly due to lack of support and education on what to expect long-term and limited follow-up care.

**Meaning:**

Results of this study suggest that coordination, physician and patient education, and mental health support are gaps in care for survivors of SJS/TEN that need to be addressed for better health outcomes and building trust between patients and their physicians.

## Teaser

This qualitative study reports on mental and physical health effects and potential gaps in care of survivors of Stevens-Johnson syndrome and toxic epidermal necrolysis.

## Introduction

Stevens-Johnson syndrome and toxic epidermal necrolysis (SJS/TEN) are rare, life-threatening, drug-induced severe cutaneous adverse reactions.^1,2^ Beyond acute morbidity and mortality, SJS/TEN causes visual impairment, blindness, and long-term effects impacting functioning and quality of life.^3,4^ Stevens-Johnson syndrome and toxic epidermal necrolysis affect 1 to 5 million persons per year.^5,6,7^ These unpredictable reactions occur in patients who may otherwise be healthy, creating major challenges during illness and after discharge.^8,9^ While acute impacts are well studied, there is a clear need for more research on long-term physical, psychological, and quality-of-life outcomes.^10,11^

Patients with SJS/TEN often feel supported in the hospital, particularly at specialist centers.^12^ After discharge, however, most report inadequate support and care as they manage chronic physical and psychological symptoms, creating stress for both patients and caregivers.^12,13,14,15^ As with other rare diseases, long-lasting psychological impacts, including alienation, poor self-image, and impaired coping, further reduce quality of life.^5,13,16,17,18^

Survivors of SJS/TEN report fragmented care for comorbidities often worsening after diagnosis.^13,14^ Most do not receive counseling on safe future medication use.^15^ These gaps lead to physician distrust and fear of medications.^12^ With limited physician knowledge, patients and families frequently turn to internet resources, and support groups exist for the many patients who are not connected at diagnosis or predischarge.^19,20^ To our knowledge, this is the largest US qualitative investigation of survivors of SJS/TEN after discharge examining gaps and unmet needs in the US health care system.

## Methods

### Sampling and Recruitment

This qualitative investigation used semistructured phone interviews with study personnel trained in qualitative methods (K.R.B., MA in Social Psychology, Vanderbilt University Qualitative Research Core Senior Research Manager, female, 13 years’ experience, no prior relationship with study participants). Participants were informed of the study’s purpose and that the interviewer was a trained qualitative researcher with no clinical background and in line with prior qualitative research studies.^21^ Participants were chosen from within a community-based study and had experienced drug-induced SJS/TEN. The SJS Survivors Study was approved in August 2019 by the Vanderbilt University Medical Center’s Institutional Review Board (IRB No. 191350). A cross-sectional cohort of enrolled participants with different lengths of stay following acute illness were invited to participate in voluntary phone interviews and were selected to provide diversity of age, time since reaction, drug culprit, gender, and race and ethnicity. Race and ethnicity for participants were ascertained through self reporting on study consent. All participants gave verbal consent to be interviewed and to have their quotations used in a publication.

### Data Collection

Interviews took place from July 2021 through August 2023. Interviews were scheduled once participants had completed the study that preceded these interviews. A semistructured interview was developed by the coauthors in collaboration with the Vanderbilt University Qualitative Research Core. The guide had 6 major questions (Supplement 1), with additional probing questions, taking participants from the start of their symptoms and hospitalization through the long-term mental and physical effects of their SJS/TEN reaction. The average duration of each interview was 45 to 60 minutes. The phone interviews were recorded for transcription purposes. All identifying data were changed or removed to ensure confidentiality.

### Data Analysis

Qualitative data coding and analysis were managed by the Vanderbilt University Qualitative Research Core members. Data coding and analysis were conducted by following the Consolidated Criteria for Reporting Qualitative Research (COREQ) guidelines, an evidence-based qualitative methodology.^22,23,24^

A hierarchical coding system was developed and refined using the interview guide and a preliminary review of the transcripts (Supplement 1). To establish intercoder reliability, 3 experienced qualitative coders independently coded 2 transcripts. Coding was then compared, and all discrepancies resolved through team reconciliation discussion.^25^ After establishing reliability using the coding system, the remaining 27 transcripts were divided and independently coded. Each patient response was treated as a separate quote and could be assigned up to 23 different codes. Transcripts, quotations, and codes were managed using Microsoft Excel 2016 (Microsoft Corporation) and SPSS version 28.0 (IBM Corporation). Analysis consisted of interpreting the coded quotations and identifying higher-order themes, using an iterative inductive-deductive approach.^26,27,28,29^

This approach was used to develop a conceptual framework informed by biopsychosocial theory while inductively integrating details from the coded qualitative data.^30,31^ Biopsychosocial theory suggests that biological, psychological, and social factors interact and affect a survivor’s posthospitalization trajectory. It is used to incorporate a holistic approach to studying the long-term mental and physical health consequences, examining the interpersonal consequences, and to understand how our health care system can improve care provided to survivors of SJS/TEN.

## Results

Of the 40 individuals contacted, 29 participated in an interview and are included in the final analytic sample. The participants, aged 26 to 76 years, were 66% female and 69% White and had experienced SJS/TEN from a wide range of drugs (Table). The length of time since reaction varied among participants, with a median of 6 years (IQR, 4-15 years).^32^ A total of 41% were interviewed within 5 years of their SJS/TEN reaction. A total of 76% had severe disease (≥10% of body surface area affected).

**Table.** Study Participant Demographic Characteristics

Characteristic	No.
Participants	29
Female, No. (%)^a^	19 (66)
Asian	2
Black	4
Hispanic/Latino	2
White	11
Male, No. (%)^a^	10 (34)
Black	1
White	9
Phenotype	
SJS <10% BSA	7
SJS/TEN ≥10% BSA	22
Age	
20-29 y	2
30-39 y	12
40-49 y	3
50-59 y	6
60-69 y	4
70-79 y	2
Age, mean (SD), y	46.3 (14.2)
Age, median (IQR), y	43 (36-57)
Time since reaction, y	
1-5	12
5-10	7
11-15	3
16-20	1
21-25	1
26-30	1
31-35	4
Time since reaction, mean (SD), y	11.3 (10.9)
Time since reaction, median (IQR), y	6 (4-15)
Pharmacological agent	
Allopurinol	3
Trimethoprim-sulfamethoxazole	9
Cephalosporins	1
Lamotrigine	3
Malarone	1
Penicillin	1
Other sulfa drug	2
Sulfasalazine	4
Carbamazepine	1
Bupropion	1
Ocular involvement	
Yes	22
No	7
Genital involvement	
Yes	15
No	14

Abbreviations: BSA, body surface area; SJS/TEN, Stevens-Johnson syndrome and toxic epidermal necrolysis.

^a^ Race and ethnicity for participants were ascertained through self reporting on study consent and noted by sex given that there were almost double the number of female to male participants.

The biopsychosocial trajectory of a survivor of SJS/TEN goes through 3 distinct stages: acute, recovery, and adaptation (Figure). The acute to recovery phase includes the traumatic response that occurs during the acute reaction of SJS/TEN. Using the conceptual framework, our study identifies dynamics discussed by patients during their recovery and adaptation stages.

**Figure.**
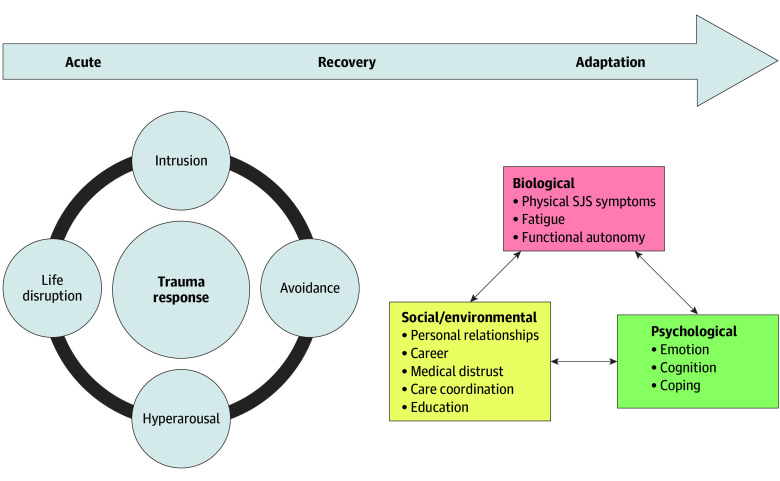
Using a Biopsychosocial Framework to Understand a Patient’s Health Trajectory Postreaction SJS indicates Stevens-Johnson syndrome.

### Biological

After being discharged from the hospital, participants discussed the range of physical symptoms they experienced during the first 6 months of recovery. These symptoms were related to known sequelae, such as skin, vision, and internal pain across different anatomical regions of the body. Vision impairment caused by SJS/TEN can be particularly life-changing and debilitating. Thirteen survivors experienced vision impairment.

…And honestly, the constant struggles with vision hasn’t been the greatest, because they’ve been definitely setbacks, especially these last couple cornea transplants and everything. So I’m used to hearing bad news, put it that way, if that makes sense. It’s just like I said before, the constant letdowns are the worst part, especially since I had the first cornea transplant and I could go back to work. I worked for a little bit of time, and that was great. (Participant 11 [male]; adaptation)

During both recovery and adaptation phases of SJS/TEN, there were numerous chronic physical changes that created challenges to patients’ mental and physical functional status and independence. Eight survivors expressed the profound importance and value of functional autonomy to their overall well-being.

…I have no quality of life right now. I can’t drive anymore because of my eyesight and my seizures. I’m not even 60. And I have to depend on people to go anywhere. And that bothers me because I’ve been very independent all of my life. (Participant 12 [female]; adaptation)

Survivors described the long-term health consequences of SJS/TEN and the difficulty of navigating tasks of daily living. Seven survivors experienced such extreme fatigue that it impacted their ability to navigate activities of daily living.

Still, almost a year later, I’m definitely not the same person, the same energy level I was before all this…in September and into October I would take a nap every day at lunch. I couldn’t make it through a workday. (Participant 1 [female]; adaptation)

### Psychological

Survivors provided considerable discussion about trauma and mental health effects during early recovery. Some were anxious and afraid when they left the hospital because they did not feel prepared. Most needed emotional support following discharge.

It’s just, my mental health while I was staying at home was probably the worst that’s been in my life.... And it was during a time where we didn’t have any contact with anyone else. So, I didn’t really have an outlet to tell anyone about what was going on. (Participant 15 [female]; recovery)

During early recovery, survivors described a preoccupation with health, misinterpretation of symptoms, and engaging in excessive internet searches. Untimely advice was a source of anger for 7 survivors.

The one piece of advice that she [physician] gave me, because I told her I was fearful to take anything above Tylenol, she said, “If you’re in a car accident, do you never get into the car again?”… I was in such a fragile state mentally, that was not the right thing to say to me. That did not resonate well. I was angry. (Participant 24 [female]; recovery/adaptation)

Some survivors experienced continued hypervigilance and obsessive worry about symptoms. Cognitive challenges were frustrating and frightening. These ongoing fears added to the burden survivors face during the adaptation phase of SJS/TEN. Five participants ended up clinically diagnosed with posttraumatic stress disorder (PTSD) and another 10 participants discussed, at minimum, some of the symptoms commonly associated with PTSD. Participants described having anxiety, intrusive and avoidant thoughts, and recurring nightmares. One common symptom of PTSD is the struggle to form meaningful connections. Six survivors with PTSD found it challenging to relate to others, triggering flashbacks.

Mental health support was a substantial source of healing for patients in recovery and adaptation. Without mental health support, survivors struggled to find healthy coping mechanisms. Others reflected that mental health referrals and support should be a mandatory posthospital discharge protocol.

Do I think that I should have had a mandatory referral to somebody for my mental health? Absolutely. I think that is something that needs to become a normal practice.… I don’t think I should have been the one to have to advocate for my mental health. I didn’t have to advocate to go see somebody to get my eyes checked after I was almost blind, so why do I have to advocate for mental health? It just should have been assigned to me. (Participant 24 [female]; acute/recovery)

### Social and Environmental

After experiencing SJS/TEN, survivors shared how their interpersonal relationships were affected. These changes were often a result of secondary trauma. For example, 1 survivor’s marriage ended because her husband could not cope with her PTSD. Four survivors expressed a need for family members to be educated and supported.

Yeah, the big thing was he [former spouse] doesn’t understand the mental health aspect of wellness…that’s probably the person whose relationship it affected the most. (Participant 24 [female]; recovery)

The inability to find a marriage partner after experiencing severe injuries led to feelings of disappointment.

One of the hardest things is I’m still wrestling with, but I’m, I’m getting better is just that here I was perfectly healthy and I never really got the, I never, never really was able to get, find someone to be married and, and to raise, a family and get my, my parents grandkids and things like that. And for a long period of time, that really bothered me. (Participant 13 [male]; adaptation)

Three individuals experienced a lack of social support alongside physical health issues, which led them to feelings of depression.

… I was abandoned. I literally don’t have any friends anymore. Everybody just sort of disappeared. It was when I was really sick, everybody was checking with my daughter. And then once I got out of the hospital, it was like poof, everybody was gone. (Participant 29 [female]; recovery)

In contrast, other survivors shared how the social support from friends, family, and social media groups played a notable role in helping them heal from the traumatic SJS/TEN event.

I would just tell them to identify the people in their life that they are 100% confident in receiving that support and not be afraid to lean on them when you need them and going through the SJS such an isolating experience that it’s really necessary to have a good support system to transition into going back into your normal routine. (Participant 15 [female]; adaptation)

Survivors shared their experiences regarding the impacts of SJS/TEN in their professional lives, with 14 having their careers impacted by their reaction. One survivor lost her job because her vision was severely impaired from SJS/TEN. Some even faced ridicule for balancing their health needs with their career responsibilities.

I’ll tell you something that was very emotional for me, which was that when I went back to work, my eyelids were starting to fuse… she (supervisor) was like, “What do you want me to do? You need to be here and work. You just took three weeks off.” That was really traumatic for me actually. (Participant 21 [female]; recovery)

Unexpected financial impacts were discussed in the adaptation phase context. During the interviews, survivors highlighted the expenses involved in managing the effects of SJS/TEN. Six survivors expressed financial frustration and strain due to their reaction and recovery.

I had to really think differently when I found out that I was going to be disabled and living on half of my paycheck and raising a son at two years old. (Participant 20 [female]; adaptation)

Following their SJS/TEN event, survivors experienced a shift in the quality of their interactions with the health care system. Less than half of participants felt that they were given clear guidance postdischarge. Inadequate knowledge and capabilities of physicians contributed to a substantial amount of anxiety and distress.

Now I do find difficulty though. I mean, there aren’t a lot of doctors who are, they may have heard about Steven Johnson syndrome or toxic epidemic neurolysis, but it’s sort of, kind of a thorough, they know that it exists or they don’t know it exists, but they don’t have any real practical experience on it. They don’t, what struggles me, what concerns me? They treat me like they would treat anybody else, but what would happen? And I want to know what I want to know what with my body, for example, I mean, I need to look out for, as I get older and I don’t think anybody can really, really tell me that. I go through one of the things in which I’ve struggled with even there is.... When you have had a reaction to a drug, in a trust, I’m still very distrusting in certain ways toward the medical field. (Participant 13 [male]; adaptation)

Navigating the health system independently was a substantial source of stress for 13 survivors, coordinating their own care without direction.

I think it’s been a major stressor to be the sole coordinator of everything. There’s not one doctor who is coordinating everything. It’s me and I’m my own advocate for this. (Participant 10 [female]; adaptation)

One survivor’s medical mistrust was so great that she went without psychiatric medication for 2 years for her preexisting psychiatric disorder after falling ill. This quote highlights that patient anxiety decreases when physicians can offer clear explanations and postdischarge guidance.

I felt like I was very much attended to.…They provided such a clear, flow diagram of what could possibly happen. What would they do if X, Y, or Z happened. And they continually had specialists coming in to check with my eyes, for ophthalmology and gynecologists and like all the necessary specialties that you needed to address when you’re dealing with SJS. So, I felt very much taken care of which definitely eased my anxiety and my overall negative state of mind at the time. (Participant 15 [female]; acute/recovery)

## Discussion

Our findings suggest that SJS/TEN has extensive impacts in survivors’ lives, even several years after discharge. Using the biopsychosocial theory as the framework, we can better grasp the complex context for recovery and adaptation (Figure). Studies have shown that the greater the psychological distress during the acute trauma of a burn injury, such as SJS/TEN, the greater the physical impairment and decreased rates of long-term recovery.^33,34,35^ This further establishes the need for the health care team to accurately assess the survivor’s biopsychosocial trajectory because the challenges they face will continue to evolve and compound on each other.^22,26^

Survivors shared their ongoing physical difficulties, including sequelae, fatigue, and loss of functional autonomy. Most physical symptoms have chronic consequences requiring multidisciplinary long-term care.^36,37,38^

Consistent with other findings, participants in this study shared emotional experiences indicative of depression, anxiety, and PTSD.^13,39^ The need for ongoing postdischarge mental health support was prevalent throughout the survivors’ interviews, aligning with previous National Institutes of Health–funded research meetings on open forums and community workshops.^6,7,10,40^

Social factors, most notably adaptation, impacted survivors’ recovery. Most affected were survivors’ career, personal relationships, and their interactions with the health care system. Survivors without social support spoke of feelings of isolation and depression. Those with mental and social support expressed more confidence. Online support groups were informative and supportive.^19,20^ Common health care system themes included the need for physician and patient education, follow-up care coordination, and a general mistrust of the system itself.

Reinforcing prior studies, a lack of education and support from their health care teams caused the survivors anxiety.^13^ A well-documented gap was the need for increased awareness and knowledge of SJS/TEN among physicians.^9,13,39^ Multiple survivors requested that sequelae-specific educational topics, including how SJS/TEN impacts potential or existing comorbidities, be discussed during hospitalization. The SJS community members proposed a holistic postdischarge educational checklist that includes short- and long-term health implications, mental health, and recovery.^41^

A concerning result of survivors’ experiences is medical distrust, driven by physician knowledge gaps, inadequate education, and the sudden unpredictable nature of SJS /TEN.^13,39^ In this cohort, this distrust led survivors to avoid necessary medications and rely on the internet for information.

Our study highlights the complexity of SJS/TEN recovery and the need for predischarge and ongoing multidisciplinary care coordination and education. Beyond physical concerns, care should address cognitive, emotional, interpersonal, and vocational challenges. Coordinated multidisciplinary support is important in a fragmented US health care system, especially since many survivors are hospitalized distant from their communities.^9,42^ Models from other rare diseases show that coordination improves outcomes.^43,44,45^ At a minimum, SJS/TEN care should include mental health, dermatology, and ophthalmology follow-up. All participants emphasized the need for postdischarge support to adapt to major life changes.

### Limitations

Limitations to our study included underrepresentation of male and racial and ethnic minority populations; 41% were interviewed within 5 years of their SJS/TEN reaction. There was potential bias as 76% had severe disease (≥10% of body surface area affected). Future studies should evaluate the standard discharge education and protocols ensuring inclusion of long-term sequelae and postdischarge mental health follow-up with trained psychologists and access to support groups.^46,47^

## Conclusions

Insights from our qualitative study of survivors of SJS/TEN may help improve understanding of long-term complications that are faced by survivors of SJS/TEN. Care coordination, physician and patient education, and mental health support are notable gaps in care of survivors of SJS/TEN. It is essential that these critical gaps are addressed with predischarge assessments and planning for better health outcomes and building trust between patients and physicians.

## Notes

Supplement 1. **eTable 1.** SJS/TEN Coding System **eTable 2.** Representative Participant Quotations Using the Biopsychosocial Approach **eMethods.** SJS/TEN Semi-Structured Patient Interview Guide

Supplement 2. **Nonauthor Collaborators.** The SJS Survivor Study

Supplement 3.
Data Sharing Statement
