# Supplementary material for: Recovering From Stevens-Johnson Syndrome and Toxic Epidermal Necrolysis
Source: JAMA Dermatol. 2025 Nov 12;162(1):24–30. doi: 10.1001/jamadermatol.2025.4345 (PMC12613091; doi:10.1001/jamadermatol.2025.4345)
Supplement: Supplement 1. — eTable 1. SJS/TEN Coding System eTable 2. Representative Participant Quotations Using the Biopsychosocial Approach eMethods. SJS/TEN Semi-Structured Patient Interview Guide [file jamadermatol-e254345-s001.pdf]

Supplemental Online Content

Martin-Pozo MD, Williams EA, Bonnet KR, et al; for the SJS Survivor Study. Recovering from Stevens-Johnson Syndrome and toxic epidermal necrolysis. *JAMA Dermatol.* Published online November 12, 2025. doi:10.1001/jamadermatol.2025.4345

eTable 1. SJS/TEN Coding System

eTable 2. Representative Participant Quotations Using the Biopsychosocial Approach

eMethods. SJS/TEN Semi-Structured Patient Interview Guide

This supplemental material has been provided by the authors to give readers additional information about their work.

© 2025 Martin-Pozo MD et al. *JAMA Dermatology*.

Table S1. SJS/TEN Coding System

Code	Label	Description	Notes/Rules
**1**	**Symptoms and Diagnosis**	**Discussion centers on the experiences of SJS/TEN symptoms and diagnosis**	
1.1	Symptoms	Discussion centers on the symptoms of SJS/TEN	
1.1.1	Skin sloughing/peeling	Skin sloughing and peeling are experienced during SJS/TEN reaction	
1.1.2	Vomiting	Vomiting is experienced during SJS/TEN reaction	
1.1.3	Rash	Rashes are experienced during SJS/TEN reaction	
1.1.4	Blisters	Blistering is experienced during SJS/TEN reaction	
1.1.5	Vision	Vision is impacted during SJS/TEN reaction	
1.1.6	Throat	Symptoms involving the throat are experienced during the SJS/TEN reaction	
1.1.7	Dizzy	Dizziness occurs during SJS/TEN reaction	
1.1.8	Bodily function	Bodily functions are impacted during SJS/TEN reaction (e.g. ability to urinate)	
1.1.9	Mouth	Symptoms involving the mouth are experienced during the SJS/TEN reaction	
1.1.10	Swelling	Swelling occurs during SJS/TEN reaction	
1.1.11	Disoriented	Participant is disoriented during the SJS/TEN reaction	
1.1.12	Flu like	Participant experiences flu-like symptoms during the SJS/TEN reaction	
1.1.13	Itchy	Participant experiences itchiness during the SJS/TEN reaction	
1.1.14	Other symptom	Other symptom not listed above	
1.2	Prompt to seek help	Reason s/he sought help	
1.3	Diagnosis	Discussion centers on experiences with the diagnosis of SJS/TEN	
1.3.1	Time of diagnosis	Discussion centered on timing of diagnosis	
1.3.2	Who diagnosed	Discussion centers on the healthcare provider who diagnosed the participant's SJS/TEN	
1.3.3	Misdiagnosis/delayed diagnosis	Discussion centers on delayed or misdiagnosis of a separate conditions.	
**2**	**Treatment experience**	**Discussion centers on patient treatment experiences and outcomes**	
2.1	Type of treatment	Discussion centers on treatment(s) received for SJS/TEN	
2.1.1	Medications	Pharmaceuticals are administered to the patient to treat and/or manage the SJS/TEN reaction	
2.1.2	Medical devices	Medical devices are used to treat and/or manage SJS/TEN symptoms	
2.1.3	Surgical	Surgical interventions are performed to treat and/or manage SJS/TEN symptoms	

© 2025 Martin-Pozo MD et al. *JAMA Dermatology*.

2.1.4	Non-pharmacological	Non-pharmacological treatments are used to treat and/or manage the SJS/TEN reaction (e.g., bandages, lotion, sunscreen, etc.)	
2.1.5	Tests	Tests are performed for symptoms associated with SJS/TEN	
2.1.6	Other type of treatment	Other type of treatment not listed above	
2.2	Treatment expectations	Discusses expectations or hopes for treatment outcomes	
2.2.1	Expectations met	Discusses treatment expectations that were met	
2.2.2	Expectations not met	Discusses treatment expectations that were not met	
2.3	Characterization of treatment outcome	Discussion centers on impact of treatment on symptoms	
2.3.1	Symptom improvement	Discusses treatment improved or eliminated of symptoms	
2.3.2	Duration of improvement	Discusses duration of symptom improvement or elimination	
2.3.3	Symptoms worsened/complications	Discusses symptoms that worsened after treatment/drug interactions	
2.3.4	Symptoms unchanged	Discusses symptoms that did not change after treatment/drug interactions	
2.4	Settings of care	Discussion centers on settings of care	
2.4.1	Outpatient	The patient is receiving care in an outpatient setting	
2.4.2	ED	The patient is receiving care in the ED	
2.4.3	Burn unit	The patient is receiving care in the burn unit	
2.4.4	Interfacility transport	Discussion centers on experiences during interfacility transport	
2.4.5	ICU	The patient is receiving care in the ICU	
2.5	Other	Other treatment experience not listed above	
**3**	**Health system interactions**	**Discussion centering on the quality of communication and interaction associated with the health system**	
3.1	Quality of interaction	Discussion centers on the quality of interactions with the healthcare team	
3.1.1	Positive health system experience	Discusses general positive experiences with health system/healthcare team	
3.1.2	Negative health system experience	Discusses general negative experiences with health system/healthcare team	
3.1.3	Limited interaction	Patient experiences limited interaction with the health system/healthcare team	
3.2	Health team qualities	Participant discusses adequacy of provider knowledge, capability to provide care, or general competence	
3.2.1	Health team knowledgeable/skilled	Discusses satisfaction with provider skills and abilities	

© 2025 Martin-Pozo MD et al. *JAMA Dermatology.*

3.2.2	Health team values patient	Provider values the patient and displays a genuine interest (e.g., provider listens to them; shared decision making; understanding of patient experience)	
3.2.3	Honesty/transparenc y/thoroughness	Provider is honest and transparent with the patient about treatment and potential outcomes	
3.2.4	Advocates for patients	Provider/health team advocates for patients (e.g., facilitates/coordinates resources)	
3.2.5	Health team care delivery failure	Patient discusses provider being incompetent or unable to deliver care as planned	
3.2.6	Health team care planning failure	Patient discusses provider being incompetent or not knowing what they are doing or being unable to create a comprehensive treatment plan	
3.2.7	Health team unknowledgeable/un skilled	Discusses a lack of satisfaction regarding the knowledge or skill of the Healthcare team	
3.2.8	Teamwork/interprofe ssional engagement	Healthcare performance centers on teamwork and interprofessional engagement	
3.3	Systems qualities	Participant discusses qualities or characteristics of a specific hospital, clinic, etc.	
3.3.1	Health Care Structures and Processes	All issues related to the organization of individuals, institutions, resources and processes for delivery of health care.	
3.3.2	Systems Economics	Discussion centers on cost and value of care	
3.3.3	Other system quality discussion	Other health system quality not listed above	
3.4	Transition of Care	Discussion centers on care transitions (e.g., transition to local centers for aftercare)	
3.5	General/other health system interaction	Other/general health system interaction not listed above	
**4**	**Interaction actor**	**The participant discusses the person whom they are interacting**	
4.1	Prescriber	Discussion is focused on interactions with the prescriber of the medication that caused the SJS/Ten response	
4.2	PCP/ongoing care	Discussion is focused on interactions with primary care/ongoing care	
4.3	Pharmacist	Discussion is focused on interactions with a pharmacist	
4.4	Dermatologist	Discussion is focused on interactions with a dermatologist	
4.5	Inpatient attending or residents	Discussion is focused on interactions with inpatient attendings or residents	
4.6	ED stae	Discussion is focused on interactions with ED stae	
4.7	EMS	Discussion is focused on interactions with EMS	
4.8	Nurse	Discussion is focused on interactions with nurses	
4.9	Social work	Discussion is focused on interactions with social workers	

© 2025 Martin-Pozo MD et al. *JAMA Dermatology*.

4.10	Family/caregiver	Discussion is focused on interactions with family and/or caregivers	
4.11	Psychologist	Discussion is focused on interactions with psychologists	
4.12	Rheumatologist	Discussion is focused on interactions with a rheumatologist	
4.13	Other interaction actor	Other interaction actor not listed above	
**5**	**Information sources/Education**	**Discussion centers on educational sources/sources of information about SJS/TEN or specific drug**	
5.1	Personal understanding of SJS/TEN	Participant discusses her/his own understanding of SJS/TEN	
5.1.1	Has some degree of understanding	Participant expresses s/he has some degree of understanding of SJS/TEN	
5.1.2	Does not understand	Participant expresses a lack of personal understanding of SJS/TEN	
5.2	Source	Discussion centers on the source of SJS/TEN information	
5.2.1	Healthcare team	Discussion centers on use of healthcare team as informational source	
5.2.2	Online sources/websites	Discussion centers on use of online sources/websites as informational sources	
5.2.3	Forums/groups/online community	Discussion centers on use of forums/groups/online communities as sources of information	
5.2.4	Family/peers	Discussion centers on utilizing family members or peers as sources of information	
5.2.5	Other	Discussion centers on using other informational source not mentioned above	
5.3	Content		
5.3.1	Drugs to avoid	Patient expresses learning about which drugs to avoid regarding SJS/TEN	
5.3.2	Potential side eeects	Patient expresses learning about the potential side eeects regarding SJS/TEN and treatment	
5.3.3	Other content	Other information content not listed above	
5.4	Useful	Discussion centers on content learned being useful	
5.5	Not useful	Discussion centers on content learned not being useful	
5.6	Information needs/wish had known	Discussion centers on information needs- what participants need to know more about	
5.7	Was not warned about SJS/TEN potential side eeect	Patient expresses a lack of warning from any source about SJS/TEN potential side eeects	
**6**	**Living with SJS/TEN**	**Discussion centers on participant's experiences living with SJS/TEN**	

© 2025 Martin-Pozo MD et al. *JAMA Dermatology.*

6.1	Mental health/emotional wellness	Discussion centers on how SJS/TEN impacts mental/emotional well-being. Also code for post-traumatic stress disorder (e.g., nightmares, unwanted memories, avoidance of situational cues, hypervigilance)	
6.2	Relationships	Participant describes how SJS/TEN impacted their ability to engage socially/within relationships	
6.2.1	Romantic	Participant describes impacts on romantic relationships following the SJS/TEN reaction	
6.2.2	Friendships	Participant describes impacts on friendships following the SJS/TEN reaction	
6.2.3	Family	Participant describes impacts on family relationships following the SJS/TEN reaction	
6.2.4	Co-workers	Participant describes impacts on relationships with coworkers following the SJS/TEN reaction	
6.3	Interactions	Participant describes how SJS/TEN created a unique interaction with others	
6.3.1	Reactions from others	Participant describes any form of reaction from another regarding their SJS/TEN	
6.3.2	How explains situation to others	Participant describes having to explain to another anything regarding their SJS/TEN	
6.4	Adjustment to life with SJS/TEN	Participant describes how they had to adjust their life to cope with SJS/TEN	
6.5	Quality of life	Discussion centers on the patient's quality of life following the SJS/TEN reaction	
6.5.1	Social isolation	Participant describes social isolation caused by SJS/TEN	
6.5.2	Autonomy	Discusses impacts on autonomy (e.g., how SJS/TEN has aeected their ability to engage with activities of daily living)	
6.5.3	ADL	Discussion centers on activities of daily living (e.g., getting dressed, shopping, chores, etc.)	
6.5.4	Work/career	Participant describes how SJS/TEN impacted their career	
6.5.5	Economic/financial	Participant describes financial/economic impact of SJS/TEN	
6.5.6	Hobbies/recreation/commitments	Participant describes how SJS/TEN impacted their ability to participate in hobbies and recreation	
6.5.7	Planning	Discussion centers on the complexity of planning activities and commitments	
6.5.8	Medical Interactions	Discussion centers on handling interactions with medical providers after hospital discharge	
6.5.9	Other quality of life	Other quality of life impacts not listed above	
6.6	Health impacts	Discussion centers on ongoing health impacts resulting from the SJS/TEN reaction	
6.6.1	Energy/fatigue	Participant experiences low energy and fatigue following the SJS/TEN reaction	

© 2025 Martin-Pozo MD et al. *JAMA Dermatology*.

6.6.2	Pain	Participant experiences pain following the SJS/TEN reaction	
6.6.3	Sexual function	Sexual function is impacted by the SJS/TEN reaction	
6.6.4	Vision	Vision is impacted by the SJS/TEN reaction	
6.6.5	Photosensitivity	Participant experiences photosensitivity following the SJS/TEN reaction (skin and/or eyes)	
6.6.6	Sleep	Participant's sleep quality and/or patterns are altered following the SJS/TEN reaction	
6.6.7	Skin Damage	Participant experiences skin damage as a result of the SJS/TEN reaction	
6.6.8	Impact on other health conditions	Participant's comorbid conditions are impacted by the SJS/TEN reaction	
6.6.9	Other health impact	Other health impacts not listed above	
6.7	Self Advocacy	Discussion centers on advocating for one's health following the SJS/TEN reaction	
**7**	**Emotions**	**Discussion involves specific feelings/emotions**	
7.1	Anger/frustration	Discussion centered around feeling frustration or anger	
7.2	Sadness/depression	Discussion centered around feeling sad or depressed	
7.3	Fear/anxiety/stress	Discussion centered around experiencing anxiety, fear, or worry	
7.4	Shock/confusion	Discussion centered around experiencing surprised or shocked	
7.5	Resentment	Participant expresses feelings of resentment	
7.6	Uncertainty	Discussion centered around feeling uncertainty	
7.7	Trust	Discussions centered around feeling trust	
7.8	Mistrust	Discussions centered around feeling mistrust	
7.9	Positive	Discussion centered around feeling gratitude	
7.10	Other	Discussion centered around another emotion not mentioned above	
**8**	**Coping Strategies**	**Discussion centers on coping strategies utilized by participants**	
8.1	Problem based	Resolve a problem to taking action to make changes or find a solution	
8.2	Emotion based	Utilizes emotion-based coping strategies such as positive thinking, pleasant activities, or other things to soothe or improve negative aeect	
8.3	Avoidant/maladaptiv e	Uses avoidance as a form of coping- e.g., intentional distraction, disengagement, substances	
8.4	Faith-Based	Utilizes religion/spiritual/faith-based strategies such as praying, attending services, meditation etc.	
8.5	Situation modification	Discussion centers on situation modification (e.g., the patient switched doctors after a negative experience)	
8.6	Peer/family support	Discussion centers on utilization of peers/social support to cope	
8.7	Psychological support	Discussion centers on use of Psychological support and resources	

© 2025 Martin-Pozo MD et al. *JAMA Dermatology.*

8.8	Other	Other coping strategy not listed above	
**9**	**Attitudes and Beliefs**	**Discussion centers on participant attitudes and beliefs**	
9.1	Normative beliefs	Discussion centers on participant's view on her/his role in a given experience or outcome	
9.1.1	Descriptive normative belief	Beliefs or perception of those around you; perception of what other people are doing (e.g., their motives)	
9.1.2	Injunctive normative belief	Beliefs or perception of what is expected of you and expected of others	
9.2	Behavioral beliefs/attributions	Discussion centers on causal attributions related to a given situation or condition	
9.2.1	Internal behavioral belief	Belief or perception that health condition or experience is the result of one's own values, intentions, or actions	
9.2.2	External behavioral belief	Belief or perception that health condition or experience is the result of situational forces	
9.3	Self-eeicacy/control beliefs	Discussion centers on patient's belief in her/his own capacity, capability, and control to engage/execute a given task	
9.4	Comparison	Any comparison made (e.g., comparison to others in same situation or dieerent situation; comparison of treatments; comparison of conditions)	
9.5	Self-perception	Way that participant views her/himself (e.g., self-image, personality, dispositional traits)	
9.6	Anticipated outcomes	Discussion centers on how behaviors/events lead to expected outcomes	
9.7	Other attitude or belief	Other attitude or belief not listed above	
**10**	**Barriers/facilitators**	**Participant describes barriers, challenges, or facilitators to SJS/TEN care**	
10.1	Barriers/challenges/deterrents	Any event/situation that lead to a breakdown in a process	
10.2	Facilitators/incentives	Discussion centers on factors that facilitate SJS/TEN care	
10.3	Contingency/conditional	Participant's response is contingent on a given factor	
**11**	**Medication(s)**	**Medication associated with the SJS/TEN reaction**	
11.1	Bactrim	The cause of the SJS/TEN reaction was the sulfa antibiotic, Bactrim	
11.2	Lamotrigine	The cause of the SJS/TEN reaction was Lamotrigine	
11.3	Other/unspecified sulfa drug	Any other sulfa drug that is not Bactrim	
11.4	Other/unspecified epilepsy drug	Any other anticonvulsant drug other than Lamotrigine	
11.5	NSAID	The cause of the SJS/TEN was an NSAID	
11.6	Unsure	Participant is unsure which drug cause the SJS/TEN reaction	

© 2025 Martin-Pozo MD et al. *JAMA Dermatology*.

11.7	Reason for drug	Participant describes the reason they were taking/prescribed the medication that caused the SJS/TEN reaction	
11.8	Other	Other medication not listed above	
**12**	**Timeframe**	**How the current discussion relates temporally to SJS/TEN**	**Designated column to identify the timeframe for each quote**
12.1	Before SJS	Discussion context focuses on life before SJS/TEN	
12.2	Time of reaction	Discussion context centers on the timing of the SJS/Ten reaction	
12.3	During transit to hospital	Discussion context centers on transit to the hospital	
12.4	Duration of stay	Anytime participant mentions length of hospital stay	
12.5	During hospitalization	Discussion context is centered on events that occurred during hospitalization	
12.6	Hospitalization Discharge	Discussion centers on the hospital discharge process	
12.7	Readmitted to Hospital	The participant was readmitted to the hospital after SJS/TEN inpatient treatment	
12.8	Post-hospitalization first 6 months	Discussion of life during the first 6 months post-hospitalization	
12.9	Post-hospitalization current	Discussion of life post-hospitalization current/after 6 months post-discharge	
12.10	Mixed timeframe	Discussion mixes timeframe (e.g., referring to before and after in the same word unit)	If mixed timeframe, include the multiple timeframes within the coding columns but make sure the mixed is indicated in the designated timeframe column
12.11	Undetermined timeframe	The timeframe of discussion is unclear	
**13**	**Participant characteristics**	**Discussion centers on characteristics of participants**	
13.1	Age	Discussion of participant age	
13.2	Income/SES	Discussion of income or socioeconomic status	
13.3	Insurance status	Discussion centers on insurance status	

© 2025 Martin-Pozo MD et al. *JAMA Dermatology*.

13.4	Home/community environment	Discusses centers on participant's home or community environment	
13.4.1	Community characteristics	Discusses home community characteristics or location (such as living in a rural or urban environment)	
13.4.2	Household characteristics/dynamics	Discusses household members such as family, descriptions of household, working remotely	
13.5	Comorbidities	Discussion centers on comorbidities of participant	
13.6	Family health history	Discussion centers on family health history	
13.7	Other demographic	Any discussion of participant characteristics not listed above	
**14**	**Notable quotes**	**Quotes that stand out as having especially noteworthy content**	
**15**	**World events**	**World events such as COVID-19 or political climate**	
**16**	**Change over time**	**Discussion centers on a change over time**	
**17**	**Suggestions**	**Participant describes needs or suggestions**	
**18**	**Advice to others**	**The participant's response to the question the interviewer asked about what advices s/he would give to another patient who just experienced SJS/TEN.**	
**19**	**Greatest frustration/bothers the most**	**Discussion centers on the greatest frustration surrounding the SJS/TEN interactions with others and/or treatment.**	
**20**	**What docs need to know**	**Participant describes specific things that doctors need to know about SJS/TEN**	
**21**	**Cannot recall experience**	**Participant cannot recall a given experience**	
**22**	**Not Experienced**	**Participant did not experience a given topic (e.g., common SJS/TEN symptoms that were not experienced)**	

© 2025 Martin-Pozo MD et al. *JAMA Dermatology*.

Table S2. Representative participant quotations using the biopsychosocial approach.

Biopsychosocial Framework	Category	Representative Quotations
Biological	Physical SJS Symptoms	*My skin, the burns. My mouth hurt so much. My eyes, I couldn't see. I couldn't go to the bathroom. I couldn't pee without blood. It was everything. My nose, because that's something that nobody ever talks about, is what does it do to your sinuses? What does it do to your nose? My nose still hasn't been right. (Participant 5 (Female); Recovery)*<br><br>*I think it was at the second follow-up, which was like a week later. All of my nails fell off, all the skin for everything. I mean, I was laying on the floor in the bathroom at home, staring at like a piece of skin that looked like half of my foot. I mean, everything came off. It was just like a snake, like someone shedding me a lot. (Participant 10 (Female); Recovery)*<br><br>*Once the blisters started going away in my mouth, I felt a hundred percent better. That was actually the worst were the blisters in my mouth, in my lips. (Participant 28 (Male); Recovery)*<br><br>*So the biggest thing is that my eyelashes, I grew a second row of eyelashes on the top and the bottom. And they actually grow inward towards my eyeball, which scratches against my retina. So I basically wear contacts 24/7 to protect my eyes from my eyelashes, and also just to avoid the pain of literally having your eyelashes in your eyes all day. (Participant 14 (Female); Adaptation)*<br><br>*My eyelashes were giving me problems with my corneas and then they would get infected. Consequently, due to that, is where I lost the cornea of my right eye. (Participant 9 (Male); Adaptation)*<br><br>*I remember my first follow-up appointment, it was a week after I was discharged, I think it was a week or two, I forget. But, I had noticed that I was having pain in my vulvar area. And I had gotten my period and I tried to use a tampon. And it was just excruciating taking the tampon out. (Participant 10 (Female); Recovery)*<br><br>*I would say the most frustrating symptom was my vagina was immensely itchy and it wasn't a yeast infection. I think it was just the skin there that was so irritated. And I think that part didn't get quite enough attention. And felt itchy inside, but it was really the skin folds that were so frustratingly itchy. (Participant 16 (Female); Recovery)*<br><br>*I wake up every night I wake up and my mouth is as dry as a bone. And, I drink, I mean, I try to keep myself hydrated. I mean, I'm not sometimes I guess dehydration is something I'm always trying to fight. But if I drink too much, then I'm spending my time going to the bathroom all the time. (Participant 13 (Male); Adaptation)*<br><br>*And honestly, the constant struggles with vision hasn't been the greatest, because they've been definitely setbacks, especially these last couple cornea transplants and everything. So I'm used to hearing bad news… the constant letdowns are the worst part , especially since I had the first cornea transplant and I could go back to work. I worked for a little bit of time, and that was great. (Participant 11 (Male); Adaptation)*

© 2025 Martin-Pozo MD et al. *JAMA Dermatology*.

	Fatigue	*I still almost a year later I'm definitely not the same person, the same energy level I was before all this but in September and into October I would take a nap every day at lunch. I couldn't make it through a workday and I was lucky I'm still working from home but I didn't have the energy and I would sit down right at 5:00 the day is over, and just lay on the couch the rest of the day because I had no energy (Participant 1 (Female); Adaptation)*

© 2025 Martin-Pozo MD et al. *JAMA Dermatology*.

		My self-esteem. Just everything. Everything. The drive that I had back then, I still have drive, but I have reservations, too. You know, I don't want to do this sometimes because my mental, it's just not there. And then I get tired quick. So it's just a lot of effects that sums up in your self-esteem because you tend to go back to what happened to you. (Participant 20 (Female); Adaptation)<br><br>*I was mentally and physically exhausted for months and it's just really hard to get healthier both mentally and physically when you're mentally and physically drained. (Participant 24 (Female); Recovery)*<br><br>*Within the first six months I definitely felt, I just felt the dryness in my eyes, the dryness in my skin. I felt like I was having chronic fatigue. (Participant 15 (Female); Recovery)*<br><br>*I definitely get tired a lot more faster so at the end of the day, I don't really have the energy to cook. I'm just like, what can we order for delivery because I'm just too exhausted…(Participant 1 (Female); Recovery)*
	Functional Autonomy	*I'm going to do a white cane walking program with them, because what bothers me the most is that I cannot leave the house and do anything without help. So I'm trying to learn how to do things independently, so I don't feel trapped as much. I feel trapped right now. I can't just hop in the car and go. I can't go walk outside by myself. And I'm trying to find ways to get around that. (Participant 29 (Female);* Adaptation)<br><br>*I thought I was scot-free coming home. I thought that after I healed and all of that, I was going to live a perfect life, but it hasn't been that way. It's been a real ordeal because I have no quality of life right now. I can't drive anymore because of my eyesight and my seizures. I'm not even 60. And I have to depend on people to go anywhere. And that bothers me because I've been very independent all of my life. (Participant 12 (Female); Adaptation)*<br><br>*I couldn't work for like five years, especially with all the eyelid and eye surgeries and everything else like that, so it was definitely a struggle for that. My parents helped me pay bills, but of course, it only goes so far. … I still had to have help with everything. Even sometimes with simple things of putting something in the microwave to cook. I couldn't really do it. Or going to the store or just driving. Or reading a box of cereal. I couldn't do it. Yeah, paying bills, I couldn't do it. Basically, I went from being completely independent to completely dependent on somebody else, especially for a while. (Participant 11 (Male); Adaptation)*<br><br>*I didn't cope with it very well. Still not coping with it very well, if I'm completely honest. I do everything I can as independently as I can, but it is a daily struggle for me. I'm angry and sad and depressed every day, because of the loss of... I can't do anything. If I want to get in the car and just go to the store, I can't do that. It's a struggle. I try to do everything that I can as independently as I can, and it frustrates me when I need help from other people to do things. And my mother actually and my daughter talked to me about asking for help. And I do sometimes, but I don't want to sometimes, just because it pisses me off basically. (Participant 29 (Female); Adaptation)*

© 2025 Martin-Pozo MD et al. *JAMA Dermatology.*

Psychological	Cognition	*Sometimes the things they'll say, the things that they're suggesting or insinuating, makes me think about what I also went through in the hospital in my own experiences. So, things like that will trigger flashbacks. Sometimes I feel like it's hard to relate to other people or it's hard for them to relate to me, but I can relate to them on a magnified scale in terms of pain they went through or things that they have experienced. (Participant 22 (Female); Adaption)*<br><br>*I got flashbacks more closer to the event, like at that time, in that year, in that two years. I wouldn't call them necessarily flash backs now but I get... I'm not physically there. Well, maybe, sometimes I can't really differentiate between the two. They're not as bad as they were. (Participant 10 (Female); Recovery/Adaptation)*<br><br>*I remember when I first got out, I was afraid to go to sleep because I thought I wasn't going to wake up. I thought I was going to die at my sleep. Isn't that weird? I mean, when you're in the hospital like that and you're in intensive care, it just gets weird. Then I remember waking up in the middle of the night feeling just having kind of panicky stuff. I remember being afraid to close my eyes. (Participant 23 (Female); Recovery)*<br><br>*Emotionally, it was a big blow. I really had no idea how to process everything. I still didn't... I knew it was real, but I didn't see it as real. I was thinking in my head, "If I go home, everything's going to be better, everything's going to go back to normal. I'll be able to see and move around and everything's fine." But of course, it is not what happened. So I think I was a bit in denial. (Participant 29 (Female); Recovery)*<br><br>*Initially, I was on this mission to find out what drugs I can and can't take. Then I let it go a little bit. I felt like I just had to let it go because I was getting too obsessed with it. (Participant 16 (Female); Recovery)*<br><br>*Mentally, I felt like I was turning into a hypochondriac. I was scared of taking pills. I was over analyzing every little symptom that was happening. I was, even when I was in the hospital, I remember having a lot of trouble swallowing food, and I actually continue to have troubles swallowing food post discharge, even up until like six months after I was discharged. Cause I think it's that, that memory never left. Feeling like I was scared to choke on food or something. (Participant 15 (Female); Recovery)*<br><br>*Now I have medical PTSD. Thank you very much. Now I'm just afraid to go back and have this ever happened to me again. It makes me hyperventilate when I think I'm getting an outbreak. It makes me crazy when I think I'm... It just makes me crazy. (Participant 5 (Female); Adaptation)*

© 2025 Martin-Pozo MD et al. *JAMA Dermatology*.

	Emotion	*I still go through depression. I don't have any support that I need to continue to really heal from a lot of things. Not only that life situation that you dealing with, but you know, when you have health issues as well, it's even worse. Just holding on. And I stand on my faith and keep me grounded. Faith is what helps me get through. (Participant 20 (Female); Adaptation)*<br><br>*I think in particular, within the first two years afterwards, I would just cry at how grateful I was to eat something or to be able to look at something or to be able to go for a walk or to be able to work. A lot of mixed with like gratitude and then just anger and frustration and resentment. (Participant 10 (Female); Adaptation)*<br><br>*It just makes you more anxious. I'm [in my 60s], it would be nice to be getting into my '70s where I didn't have to be anxious about it. Every little thing that goes wrong with me. Normally people in their '70s are concerned about their health, but not like I am. It's crazy. I don't know how you get past it because I don't think you ever really get over it. It's just, I guess my main song here. (Participant 5 (Female); Adaptation)*<br><br>*So that part, when you go through something like that, it's traumatic, and I still have eye involvement. So I'm still reminded when I look in the mirror and my eyelid looks droopy, or when I just have my passport photo taken, and no matter how I try in photos, my right eye always looks a little droopy. Unfortunately, you never can quite put it behind you. Every time I see my eye doctor, she's like, "Well, your eyes are doing remarkably well, but you know, your right eye." All of my tear ducts in my right eye are completely scarred over. (Participant 23 (Female); Adaptation)*<br><br>*All I have to do is look at my body and I cry. I'm completely scarred. I'm scarred. I have scars all over my neck and my stomach, because my chest, those are the one's that's hidden... (Participant 4 (Female); Adaptation)*<br><br>*So I would say that the way that I look really impacted my mental health. I have scaring on my face. I can't work with contact lenses anymore and I have really thick glasses. Yeah. I just look different. Then the hair falling out. So it took a lot of time to be okay with the way that I look. Now I look almost back to normal, Not completely. So that really affected my mental health. (Participant 16 (Female); Adaptation)*
	Coping	*God puts us through things that He changes for our, for our good. And he, we, we may not understand it, but He is sovereign, and He is, He's in control every aspect of our lives. (Participant 13 (Male); Adaptation)*<br><br>*It was the best thing for me (priest at hospital), because I needed to talk to somebody. Like I said before, I wasn't mad at God, but I was resentful. I felt a lot of anger, and my anger was towards the doctor…When I was talking to the priest in the hospital, I told him how I felt. And the priest was great, because he said, "Jesus asked us to forgive our enemies." And I had to process that for a while. I didn't do it right away. And then I realized it's true, the same way that he did this to me, he could use this research to save somebody else's life. So that's how I got rid of it, of my resentment. (Participant 12 (Female); Acute/Recovery)*<br><br>*But yeah, those first six months I was just so busy mentally convincing myself, ‘No, you're alive and you need to live. You need to live with purpose… (Participant 14 (Female); Recovery)*

© 2025 Martin-Pozo MD et al. *JAMA Dermatology.*

		I'm going to do a white cane walking program with them, because what bothers me the most is that I cannot leave the house and do anything without help. So I'm trying to learn how to do things independently, so I don't feel trapped as much. I feel trapped right now…. (Participant 29 (Female); Adaptation)<br><br>*I have learned lately at night in order for me to sleep, I have to write something because otherwise I can't sleep. I think I'm home all day, except for that hour that I go out walking. There's a lot of stuff in my mind. So it's almost like throwing out the garbage every day, emptying my brain…I was having problems sleeping and that's why she (therapist) told me to do that. (Participant 12 (Female); Adaptation)*<br><br>*I'd always had thought that maybe it was bad to just keep my traumatic memories under the rug and never bring it up and like just cross my fingers out for the best in my life. But I finally take it seriously and it made me realize how important it (mental health) was, how much in line it is with physical health…I think I didn't realize that I needed help and it was important for me to get it (mental health support) because it was going to change the trajectory of my life. But now I'm finally feeling the result of that. (Participant 15 (Female); Adaptation)*<br><br>*I didn't cope with it very well. Still not coping with it very well, if I'm completely honest. I do everything I can as independently as I can, but it is a daily struggle for me. I'm angry and sad and depressed every day… (Participant 29 (Female); Adaptation)*
Social / Environmental	Career	*I felt back at home, not doing much, not working, I then started to feel like, okay, I started to feel lack of purpose. Okay, what do I do now? I've worked my way up to this career and now I have to change it. And I don't know what else I want to do. So a lot of hopelessness and lack of purpose. But I would say that the counseling really helped me talk through those things. (Participant 16 (Female); Recovery)*<br><br>*So I'm supposed to avoid carbamazepine. That is another big one that can result in SJS. They gave me a laundry list of drugs, but they all treat things that I wouldn't really have to worry about until later, like thyroid disease and things like that. And then for my master's program I actually went into pharmacogenomics in response to the experience that I had with SJS. (Participant 14 (Female); Adaptation)*<br><br>*My office was wonderful to me. I took all of my accumulated sick leave, and this is important. This is what we should tell people, sick leave and vacation. So that covered, I didn't work for like three months, I think. (Participant 23 (Female); Recovery)*<br><br>*I'll tell you something that was very emotional for me, which was that when I went back to work, my eyelids were starting to fuse. I realized at the beginning of the work day and I'd already been out for two or three weeks, and I realized my eyelid was fusing and that was a new development. I need to go see the ophthalmologist because then they were going to put an amniotic membrane in my eye if that was happening. And I told my chief resident and she was like, "What do you want me to do? You need to be here and work. You just took three weeks off." That was really traumatic for me actually. She was a medical person but she didn't have any empathy after me being out for three weeks. (Participant 21 (Female); Recovery)*

© 2025 Martin-Pozo MD et al. *JAMA Dermatology*.

		And then just as I went through that (hospitalization), okay I can't do my job anymore. What am I going to do? When I came home, I was off work for five months. My boss was like, we're happy to accommodate you in any way possible, but we can't keep you on the project anymore. We are finding someone else to go there. And that was it. It was very difficult. And then watching the person who replaced me do my job was also very, very difficult. And I felt the most emotional when I was found out the person would be going to Uganda. I was like, oh, I want to go there. I want to be there. Yeah, it sucked to be sitting at home (Participant 16 (Female); Recovery)
	Personal Relationships	*I was really woozy for maybe like the first five days or so, just kind of assessing little things happening. And I remember being really mad at my husband because he took my daughter back-to-school shopping, because that was what I do. I thought, "They couldn't have waited for me?" I was really mad about this. I was still clueless. I had no idea that this could've been fatal. I was just like, "Man, y'all just kept living without me. That's rude." (Participant 14 (Female); Recovery)*<br><br>*My boyfriend at the time, he ended up cheating on me while I was in the hospital. Honestly, I think that was more traumatic than some of the things that I went through with TENS, too. (Participant 22 (Female); Recovery/Adaptation)*<br><br>*SJS is such an isolating experience that it's really necessary to have a good support system to transition into going back into your normal routine. And I just feel that it's incredibly important to be transparent with people you trust, about what you went through from start to finish and your experience and being able to show them the negative feelings that you were experiencing with that, just being 100% transparent with them and how bad things were relieves so much of the anxiety. (Participant 15 (Female); Adaptation)*<br><br>*I think it was the support from my boyfriend, honestly, because he was the one who gave me that extra push to get mental health treatment. It takes a while for someone to get to that point. And he was the helping hand to get me there. And when things were rocky... When things were rocky with my parents, he was also there to support me. So, I definitely felt like he made me feel like... normalized situation as much as he could and did everything, he could to transition into going back into normal life. (Participant 15 (Female); Recovery)*<br><br>*I wondered if there's a Stevens-Johnson one (FB group) now. And so I looked that up, but it's really bad for me to look up both of those support groups because everyone's just talking about how terrible it is and how scared they are. And so it just brings it all up. (Participant 21 (Female); Adaptation)*<br><br>*There's two (support groups) on Facebook for Stevens Johnson Syndrome that I belong to and that's helpful. I didn't find them until probably three or four weeks after I got out of the hospital. It never occurred to me to even look for that on Facebook but that definitely helps. I try to do my part too and help people that, luckily for them, found it a lot earlier than me when they have questions because I know what it's like to just feel like you're out there and clueless and you don't even know what's going to happen next. (Participant 1 (Female); Recovery)*<br><br>*Yeah, the big thing was he (former spouse) doesn't understand the mental health aspect of wellness. If you can't see it, it's not there, so I would wake up with nightmares. He would crawl into bed later than me and then touch me, and I'd freak out because I was having a nightmare at the time. I didn't know what was going*

© 2025 Martin-Pozo MD et al. *JAMA Dermatology.*

		on. I was having a flashback, whatever it was, and he chalked it up to me over reacting. Yeah. So that's probably the person whose relationship it affected the most. (Participant 24 (Female); Recovery)<br><br>*I felt like because I looked different, I had to explain to people what happened to me. Because I felt like no one was going to be like, oh, you look different. What happened to you? You know? And maybe I didn't need to, but I felt like I had to explain to people what happened and why I looked different. And I think I avoided social situations for a while. I definitely did not want to be in any pictures even. (Participant 16 (Female); Adaptation)*<br><br>*There was the women one time who were talking about having babies. So, they were saying, "Yeah, it's tough when you're in labor." Sometimes the thing they'll say, the things that they're suggesting or insinuating, it makes me think about what I also went through in the hospital in my own experiences. So, things like that will trigger flashbacks. Sometimes I feel like it's hard to relate to other people or it's hard for them to relate to me but I can relate to them on a magnified scale in terms of pain they went through or things that they have experienced. (Participant 22 (Female); Adaption)*<br><br>*I don't have social life. The only people that I can talk to, and I don't talk to them on a regular basis, are the nurses and doctors. And they understand what kind of life I'm living, people with anxiety, depression, people with pain. And they understand that. Some people, they don't understand it and they say things to piss you off. I can't talk to people like that. Even my family, I lost my family because of that. They feel that I can't talk to them. We can't talk, so I lost all my friends. (Participant 8 (Male); Adaptation)*<br><br>*I notice that I don't look people in the eyes as much anymore just because I don't like making people uncomfortable and I've noticed that I also, myself, feel uncomfortable when people ask, "Oh my gosh, are you okay? Your eyes look so red." There's a few times that people are like, "Are you high right now? You look like your eyes are bloodshot." It's just embarrassing. I don't like people feeling sorry for me, but I also just generally, you know, I avoid people because sometimes it just feels like it's exhausting. I feel like I'm so much more of an introvert now... (Participant 2 (Female); Adaptation)*<br><br>*One of the hardest things is I'm still wrestling with, but I'm, I'm getting better is just that here I was perfectly healthy and I never really got the, I never, never really was able to get, find someone to be married and, and to raise, a family and get my, my parents grandkids and things like that. And for a long period of time, that really bothered me. (Participant 13 (Male); Adaptation)*

© 2025 Martin-Pozo MD et al. *JAMA Dermatology*.

	Healthcare	**Education**<br><br>*I've done a lot of searching after I'd been discharged and was recovering. And I think the main site that I was really digging into was there was like an SJS foundation site where it had a lot of resources on support groups and other patients stories and like general information on how to take care of short term, long term effect, like physiology and stuff like that. So I'd gone through there, and I was briefly reading journal articles on SJS. Cause I just wanted to know more research brought in like what the... What the current bulk of research there was out there, but it didn't seem much. So it definitely told me that there needed to be more work on understanding SJS and just real empirical studies on it that I felt like I didn't see as much as I wanted to. (Participant 15 (Female); Recovery)*<br><br>*Now I do find difficulty though. I mean, there aren't a lot of doctors who are, they may have heard about Steven Johnson syndrome or toxic epidemic neurolysis, but it's sort of, kind of a thorough, they know that it exists or they don't know it exists, but they don't have any real practical experience on it. They don't, what struggles me, what concerns me? They treat me like they would treat anybody else, but what would happen? And I want to know what I want to know what with my body, for example, I mean, I need to look out for, as I get older and I don't think anybody can really, really tell me that. I go through one of the things in which I've struggled with even there is... When you have had a reaction to a drug, in a trust, I'm still very distrusting in certain ways toward the medical field. (Participant 13 (Male); Adaptation)*<br><br>*Looking at other people and the symptoms that they had, and coming across stories of other people and their first-hand experience, and not feeling so alone. And then reading scholarly papers, and understanding the mechanism of action that these drugs take on with these mutations that cause this cutaneous reaction. It's a little exhausting, and the scholarly route doesn't really seem to give me the answers that I want so much as the first-hand accounts do. (Participant 14 (Female); Recovery)*

© 2025 Martin-Pozo MD et al. *JAMA Dermatology*.

		I wasn't in contact with anybody that had any information that was confident enough. I went to an allergist who, honest to God, opened up his medical book, had me hold one end of the book, he had the other end of the book, and he read word for word what Stevens-Johnson syndrome was. Never gave me any information, so I was distraught...I was trying to look for answers about what now? It could have killed me. How do I live my life now? What do I have to do? I felt like if you go in and you get a pacemaker put in because you've had a heart attack, they tell you how you should be living your life, what medications you need to be taking, what you should be on the lookout for. I never got any of that. So, I left very scared, very nervous. (Participant 24 (Female); Acute/Recovery)<br><br>*It wasn't like, "Oh, we are the doctors. We know what we're talking about and you're beneath us." They were really willing to learn from us, wanting to learn from us. They were wanting to give us as much information and arm us with as much as we could, so that conference there was a game changer for me. I learned everything that I basically know about Stevens-Johnson's and what I'm supposed to avoid and that type of thing from there, so that was when things really clicked …Once you're armed with information, you can do something with it. That's what I needed. I needed the answers. I needed the information so that I knew how to go about my day-to-day, because up until that point, I had nothing. (Participant 24 (Female); Adaptation)*<br><br>*I think that more education in the hospital before discharge is most helpful. Not even just a follow up with just dermatology, but maybe it would've been helpful if I had seen an eye doctor upon leaving the hospital, but it wasn't "recommended." So I never did. But just maybe having a multidisciplinary team follow up with you so many weeks or so after discharge would've been helpful. And then lastly, the mental health piece was definitely missing, and I think anybody could benefit from that. (Participant 26; Acute/Recovery)*<br><br>*It would've been nice if they would've warned me that I would continue to lose my fingernails and toenails, and in long term, they would still crack. I mean, this month it'll be five years. Once we were out of there, we didn't know what to expect. The situation with the skin coming off the heels of my feet. We never expected anything like that. (Participant 28 (Male); Acute/Recovery)*<br><br>**Care Coordination**<br><br>*I think it's been a major stressor to be the sole coordinator of everything. There's not one doctor who is coordinating everything. It's me and I'm my own advocate for this. I've been to so many different doctors and I have noticed that to try and find the ones that are willing to do the research, even if they have it in a TEN patient before, most of cause 99% of them have it. I've definitely seen doctors who I tell them what it is and*

© 2025 Martin-Pozo MD et al. *JAMA Dermatology.*

		they just blind need stare and they say, oh, okay, and I'm like, wow, okay. Like this is not going to work out. (Participant 10 (Female); Adaptation)<br><br>*Build up your care team. Find a GP who's going to be the central organizer of your care team that is sensitive. And at least if not real knowledgeable with SJS, at least open to learn and work with you. If your primary care physician is not going to listen to you or not take you seriously, find one that will because that's the key. (Participant 5 (Female); Adaptation)*<br><br>*The after-visit summaries. I have saved every single one from every single appointment. It's not just that, it tells you what you're allergic to, what your care instructions are, if there's something else that the doctor missed, they include it on there, your next appointments. It's an invaluable resource, put it that way. (Participant 11 (Male); Adaptation)*<br><br>*Going to all these different doctors takes a lot of time. It's expensive. (Participant 10 (Female); Adaptation)*<br><br>**Medical Distrust**<br><br>*I'm a little hesitant to go back to the same doctor because the problem with Stevens-Johnson is that it doesn't hit everyone the same. So even if you've worked with someone who has Stevens-Johnson, you still don't know my situation. And the same way that if you ask a carpenter to solve a problem, the answer's always going to be a hammer and a nail, even if it's a plumbing issue. I feel like every doctor I come across wants to take my situation and relate it to something that they've already done, and say, "Yes, I know exactly how to do that," when they really don't, and then they're surprised when it doesn't work. And then it's exhausting and it's frustrating, because you go into every surgery really full of hope and then you're just disappointed again. (Participant 14 (Female); Adaptation)*<br><br>*And there was a lot of anger and there's a lot of distrust. I was a trusting individual before all of this happened. And then when I learned what the doctor, what happened from the doctor, I was so full of distrust. I didn't know, I didn't, I couldn't trust anyone. The only person I could trust was my family and the minister who was in my, at my church. (Participant 13 (Male); Recovery)*<br><br>*I guess it made me have my doubts and maybe the medical field as a whole, that seems very general, but you go to the hospital in hopes that somebody could diagnose you and fix you for lack of better words but just make you better and know right away what it is. And whenever there's confusion or a disagreement amongst the providers, I don't know, you kind of lose a little bit of trust and confidence in the hospital system. And yeah, it's not a good feeling. But I mean, I think looking back at it now, I feel very lucky that I had a somewhat mild case in comparison to some people, but even to this day, don't feel like I fully trust any providers take on what medicine could or could not be safe for me, if that makes sense. (Participant 26; Adaptation)*<br><br>*I know I was very on edge and I don't know, nobody could really console me and convince me that it was okay for me to take it. Feels like I didn't trust that it was not going to end up in the same situation. (Participant 3; Recovery)*

© 2025 Martin-Pozo MD et al. *JAMA Dermatology*.

		For the first two years, yes. I did not take any medication. Then I had gotten so depressed…this psychiatrist that I was referred to has been so, like the very first thing she said to me was, 'oh my gosh, you don't miss Lamictal and Depakote'. I was like, 'oh my goodness. I love you'. (Participant 10 (Female); Adaptation)<br><br>*I have taken it upon myself. Again, I just live in a small town, but I have reached out to our first responders…I know that it's not going to be very often that somebody in our small rural area is going to call 911 to get an ambulance if they have SJS symptoms, but maybe it'll happen, so I have talked to them to educate them, to just have it on their radar. I had reached out and nothing went anywhere with it to the hospital that I was admitted to in (state) to say, "Hey, can I come and talk to you people? I'm not here to teach you anything medicine wise. I'm here to teach you about my experience so that this can be at the back of your mind if this happens again to anybody else." (Participant 24 (Female); Adaptation)*<br><br>*It's not that I don't trust them (providers). It's just that I'm more wary about thing and particularly about what they give me. As an example, I take Advair inhaler…he also wanted me to take Albuterol as a rescue inhaler. Well, recently I was reading on the internet through Steven Johnson that they had like one or two cases that in children who had had outbreaks of Steven Johnson syndrome with Albuterol. Now, granted, it's one of the case I mean, it's rare to have that, but I feel rather fearful of taking that, because all I need is one time for, to have a recurrence of Steven Johnson. And so I'd rather not take something like that if I don't need it and I don't use it. (Participant 13 (Male); Adaptation)*<br><br>*The ENT, they're saying it's acid reflux. I never had a problem with heartburn or anything. And I still do. I get these pains in my throat that radiates down to my back, my chest. It's unbearable. I literally have to lay down. And she (ENT) was going to prescribe me omeprazole, so I said, "I'm not taking that." She said, "Why?" And I said, "Because it has sulfa in it." She said, "It does?" I was done. I was done at that. (Participant 4 (Female); Adaptation)*<br><br>*I was so fearful and I wasn't in contact with anybody that had any information that was confident enough. I went to an allergist who, honest to God, opened up his medical book, had me hold one end of the book, he had the other end of the book and he read word for word what Stevens-Johnson syndrome was. Never gave me any information, so I was distraught. I was scared. If these people don't know what to tell me, how do I know what I can and can't do? How do I know if this is going to happen again? How do I avoid this from happening again? (Participant 24 (Female); Recovery)*<br><br>*Both the psychologist and counselor did not know what it was. The reason I chose them though is because they had experience dealing with people who had undergone trauma. But I did have to explain it to them. And then in the next visit they told me that they did research it and looked into it in more detail. (Participant 16 (Female); Recovery)*<br><br>*Yeah. However, one doctor, I think the doctors who are most interested have been the eye doctors. They're most interested in, collectively as a whole …The one eye doctor I have because she works, she consults at a burn unit in New York .... and I asked her, 'Do you know of any gynecologists who [inaudible] in her burn*

© 2025 Martin-Pozo MD et al. *JAMA Dermatology*.

		unit?' She's like, 'We don't do it.' She's like, 'If you find a gynecologist, tell me so that I can tell other people.'. That's the kind of medical professional that you want. Not everyone's like that. (Participant 10 (Female); Adaptation)

© 2025 Martin-Pozo MD et al. *JAMA Dermatology*.

eMethods. SJS/TEN Semi-Structured Patient Interview Guide

Patient Study ID _________ Interview Date __________

Hello, my name is [INTERVIEWER'S NAME] from the Qualitative Research Core from Vanderbilt University. I am working with my colleagues on an important study that focuses on physical and mental health following Stevens-Johnson syndrome and toxic epidermal necrolysis (SJS/TEN). As a participant in the SJS Survivor study you would have experienced SJS/TEN associated with a medication and now be in the recovery phase of SJS/TEN.

The aim of this interview is to learn from you about the long-term health consequences of SJS/TEN to understand how we can improve the care we provide SJS survivors. I will ask a total of 6 questions and depending on the length of your answers, our discussion should take about 20 to 30 minutes. There are no right or wrong answers to these questions, we simply want to hear your perspective. You do not have to answer any questions you don't feel comfortable answering and can take a break at any time.

I would like to record our interview. This allows me to focus on you, instead of trying to jot down specific details during our discussion. Everything you say today will be kept confidential between you, me, and our research team. We are writing a report but nothing you say will be linked to you. May I record our interview? We would like to get your verbal consent to agree to take part in this voluntary discussion. (read the consent and document on REDCap) Do you agree to participate?

In order to maintain your privacy, please use first names only and avoid using any personally identifying information.

What questions do you have before we get started?

If there are no more questions let's begin the interview.

1) Do you remember being diagnosed with SJS/TEN in [month/year] at [name of hospital]?

Additional prompts

- When did it become apparent to you that you should seek medical attention?
- How far was the hospital that you were admitted from your home?
- Were you alone or did anyone accompany you?
    - Explain who and relationship

- What are some things that you remember about that experience?
    - Time of reaction
    - During transit
    - While in hospital

© 2025 Martin-Pozo MD et al. *JAMA Dermatology*.

- What do you remember most about your hospitalization? (most memorable experience)
        - Staff interaction
        - Treatment
        - Discharge

- What was this experience like for you emotionally at the time?
    - Tell me more

2) What symptoms associated the SJS/TEN concerned you the most during your hospitalization?
    a. What symptoms associated with SJS/TEN were you most concerned about during the first 6 months after leaving hospital?

Probe further
- What physical symptoms did you feel the first 6 months after leaving hospital?
- How is your vision currently? Do you have eye complications currently?
- Has your vision changed over the last six months?
- Has your vision changes since you were hospitalized for SJS/TEN?
- Were you able to sleep and eat normally?
- Did you feel anxious or depressed?
- Do you have flashbacks of your time in hospital after leaving hospital?
- Do you have panic attacks currently?
- Have you had panic attacks at any time after SJS? How long did they last for?
- Did you have adequate support?
- Who was your greatest source of support?
- How are you managing activities of daily living?
- Were you able to return to work?

3) Were you able to find doctors or healthcare professionals who knew about SJS/TEN? Other resources?
    a. Barriers
    b. Facilitators or solutions to any barriers

Probe further
- How did you feel about the support from your healthcare team during recover?
    - What went well
    - What can be better
- Did you feel that those taking care of you knew enough about SJS/TEN?
    - Ask for example
- What was your source of information about SJS/TEN?
    - Health team
    - Internet/forums
    - Family

© 2025 Martin-Pozo MD et al. *JAMA Dermatology.*

- What is your greatest frustration about any aspect of SJS/TEN or your care at any point during your acute care and follow-up?

4) What medication was associated with your SJS/TEN?
- Ask patient to name drug
    - If can't remember/do not know, ask what it was prescribed for
        - Epilepsy
        - Mood disorder
- What information was given to you about which drugs to avoid?
    - Clarity
    - What wish would have known
- How confident did doctors and your medical team seem about the information they gave you?
    - Example
- What information did you seek about the medication that cause SJS/TEN? (if independently sought)
    - Internet/forums
    - Family
    - Pharmacist
- What was the most helpful source of information?
    - What was helpful/useful about this source?
- What feelings do you have about taking drugs in the future?
- What do you wish you would have known/learned while hospitalized that you know now?

5) What has changed the most about your mental and physical health since you have experienced SJS/TEN?
    a. Physical
    b. Mental

Probe further
- In what ways do you feel that your health today is different from before your diagnosis with SJS/TEN?
- Do feel any current major medical problems are related to SJS/TEN?
    - Tell me more.
- Has SJS/TEN affected treatments for your other health conditions?
    - In what ways?
- Do you have chronic pain?
    - Please describe
- Have your sought treatments for chronic pain?
    - Which treatments?
    - Successful/unsuccessful?
- Have your sought alternative medical treatments

© 2025 Martin-Pozo MD et al. *JAMA Dermatology.*

- Successful/unsuccessful?
- What has helped the most in terms of your adjustment post-SJS/TEN?

6) Has SJS/TEN affected the way you interact with others?
    a. In what ways?
- How would you describe your general quality of life?
    - Activities of daily living
    - Hobbies/recreation
    - Work/financial
    - Emotionally
    - Travel
    - Social
- Were you in a long-term relationship at the time of SJS?
    - How has this been affected?
- What long-term effects of SJS have you had that affect your interaction with others?
    - Sexual function
    - Energy
    - Mood
    - Vision
    - Pain
    - Post-traumatic stress disorder/anxiety
    - Other physical or mental disability affecting social interactions

© 2025 Martin-Pozo MD et al. *JAMA Dermatology*.
