# Supplementary material for: Recovering From Stevens-Johnson Syndrome and Toxic Epidermal Necrolysis
Source: JAMA Dermatol. 2025 Nov 12;162(1):24–30. doi: 10.1001/jamadermatol.2025.4345 (PMC12613091; doi:10.1001/jamadermatol.2025.4345)
Supplement: Supplement 2. — Nonauthor Collaborators. The SJS Survivor Study [file jamadermatol-e254345-s002.pdf]

Supplemental Online Content: Nonauthor Collaborators
*First name, last name, and suffix (if applicable) are required and will appear in PubMed.

*Group Name(s): SJS Survivor Study							
***First Name and Middle Initial(s)**	***Last Name**	***Suffix (eg, Jr, III)**	Academic Degrees	Institution	Location (city, state/province, country)	Role or Contribution, eg, chair, principal investigator	Group (if more than 1 Group listed in the byline) and/or Subgroup (eg, Steering
Roni	Dodiuk-Gad		MD	Technion Institute of Technology	Haifa, Israel	Investigator/Adjudicator	SJS Survivor Study
Aaron	Drucker		MD, SCM, FRCPC	Womens College Hospital	Toronto, Ontario, Canada	Investigator/Adjudicator	SJS Survivor Study
Elizabeth	Ergen		MD	University of Tennessee	Knoxville, TN, USA	Investigator/Adjudicator	SJS Survivor Study
Rama	Gangula		MS	Vanderbilt University Medical Center	Nashville, TN, USA	Assistant	SJS Survivor Study
Michelle	Goh		MBBS	Austin Health	Heidelburg, VIC, Australia	Investigator/Adjucator	SJS Survivor Study
Dana	King		BS	Vanderbilt University Medical Center	Nashville, TN, USA	Assistant	SJS Survivor Study
Rebecca	Lee		MPH	Vanderbilt University Medical Center	Nashville, TN, USA	Assistant	SJS Survivor Study
Kelby	Mahan		LPN	Vanderbilt University Medical Center	Nashville, TN, USA	Assistant	SJS Survivor Study
Madeline	Marks		BS	Vanderbilt University Medical Center	Nashville, TN, USA	Assistant	SJS Survivor Study
Robert	Micheletti		MD	University of Pennsylvania	Philadelphia, PA, USA	Investigator/Adjucator	SJS Survivor Study
April	O'Connor		RN	Vanderbilt University Medical Center	Nashville, TN, USA	Assistant	SJS Survivor Study
Suman	Pakala		ME	Vanderbilt University Medical Center	Nashville, TN, USA	Assistant	SJS Survivor Study
Amy	Palubinsky		PhD	Vanderbilt University Medical Center	Nashville, TN, USA	Assistant	SJS Survivor Study
Misha	Rosenbach		MD	Penn Medicine	Philadelphia, PA, USA	Investigator/Adjucator	SJS Survivor Study
Kristina	Williams		RN	Vanderbilt University Medical Center	Nashville, TN, USA	Assistant	SJS Survivor Study
Alicia	Wright		MS, CCRP	Vanderbilt University Medical Center	Nashville, TN, USA	Assistant	SJS Survivor Study
Alexis	Yu		MPH	Vanderbilt University Medical Center	Nashville, TN, USA	Assistant	SJS Survivor Study
