# Supplementary material for: Recovering From Stevens-Johnson Syndrome and Toxic Epidermal Necrolysis
Source: JAMA Dermatol. 2025 Nov 12;162(1):24–30. doi: 10.1001/jamadermatol.2025.4345 (PMC12613091; doi:10.1001/jamadermatol.2025.4345)
Supplement: Supplement 3. — Data Sharing Statement [file jamadermatol-e254345-s003.pdf]

Data Sharing Statement

Martin-Pozo. Recovering From Stevens-Johnson Syndrome and Toxic Epidermal Necrolysis. *JAMA Dermatol*. Published November 12, 2025. doi:10.1001/jamadermatol.2025.4345

Data
Data available: No

Additional Information
Explanation for why data not available: The data underlying this article will be shared at reasonable request to the corresponding author.
